# Management of renal sinus angiomyolipoma: modified robotic nephron-sparing surgery in a single center

**DOI:** 10.1186/s12894-024-01492-x

**Published:** 2024-05-07

**Authors:** Yan Zhang, Zongbiao Zhang, Fan Li, Wei Guan

**Affiliations:** Department of Urology, Institute of Urology, Tongji Hospital, Tongji Medical College, Huazhong University of Science and Technology, No. 1095 Jiefang Avenue, Wuhan, Hubei 430030 China

**Keywords:** Robotic surgery, Nephron-sparing surgery, Renal sinus tumor, Angiomyolipoma, Experience

## Abstract

**Background:**

Renal sinus angiomyolipoma (RSAML) is a rare and typically complex renal tumor. The objective is to present our single-center experience with a modified technique of robotic nephron-sparing surgery (NSS) for treating RSAML.

**Methods:**

We retrospectively evaluated 15 patients with RSAMLs who were treated with robotic NSS at the Department of Urology of Tongji hospital, ranging from November 2018 to September 2022. Renal vessels and ureter were dissected. The outer part of RSAML was resected. The rest of tumor was removed by bluntly grasp, curettage and suction. Absorbable gelatin sponges were filled in the renal sinus. The preoperative parameters, operative measures and postoperative outcomes were all collected. Follow-up was performed by ultrasonography and estimated glomerular filtration rate (eGFR).

**Results:**

Robotic NSS was successfully performed in all the patients, without any conversion to open surgery or nephrectomy. The mean operation time was 134.13 ± 40.56 min. The mean warm ischemia time was 25.73 ± 3.28 min. The median estimated blood loss was 100 [50, 270] ml and 1 patient required blood transfusion. The mean drainage duration was 5.77 ± 1.98 days. The median postoperative hospital stay was 6.90 [5.80, 8.70] days. Two patients experienced postoperative urinary tract infection (Clavien-Dindo Grade II). During the median follow-up of 25.53 ± 15.28 months, patients received 91.18% renal function preservation. No local recurrence occurred in all the patients.

**Conclusions:**

Robotic NSS for RSAML is a complicated procedure that demands technical expertise and a well-designed strategy is critical in the operation. Treating RSAML with modified robotic NSS is safe, effective and feasible.

## Background

Angiomyolipoma (AML) is a common benign neoplastic lesion in the kidney [1, 2]. Although the majority of AMLs are asymptomatic and slow growing, 10-15% of AMLs occur spontaneous rupture which may result in hypovolemic shock in 30% of these patients [3]. Prophylactic treatments have been suggested for symptomatic AMLs or AMLs with a diameter of > 4 cm to prevent spontaneous bleeding [1].

Selective arterial embolization (SAE) is the preferred initial treatment, with a success rate of 90-100% [3]. However, it only provides a 23% reduction in the volume [4]. Re-growth and progression of the initial disease after blocking the blood supply remain a concern [5]. Laparoscopic nephron-sparing surgery (NSS) represents an alternative option for treating renal AML. Laparoscopic NSS is more invasive than SAE, but it enables complete AML resection and offers a low risk of recurrence. Recent studies report almost no recurrence of sporadic AML after laparoscopic NSS [6, 7]. Novel strategies have been developed to minimalize destruction of kidney in NSS [8–10]. Conversion to open surgery or nephrectomy is rare, and preservation of renal function could reach 83-98%.

Renal sinus AML (RSAML) is quite rare. RSAML has a deep location, occupies renal sinus, and is surrounded by collecting system and renal blood vessels. NSS for RSAML is quite complicated and technically challenging even for experienced surgeons. Robotic surgery shows great advantage in the treatment of complex renal tumors [11], because of its better movability, clearer field of view and greater stability of operation. Previous studies have shown robotic surgery for common renal AML [12, 13], but few experience for RSAML has been reported. In order to solve this problem, we retrospectively evaluated robotic NSS for RSAML and summarize our single-center experience.

## Patients and methods

### Patients

**Fig. 1:**
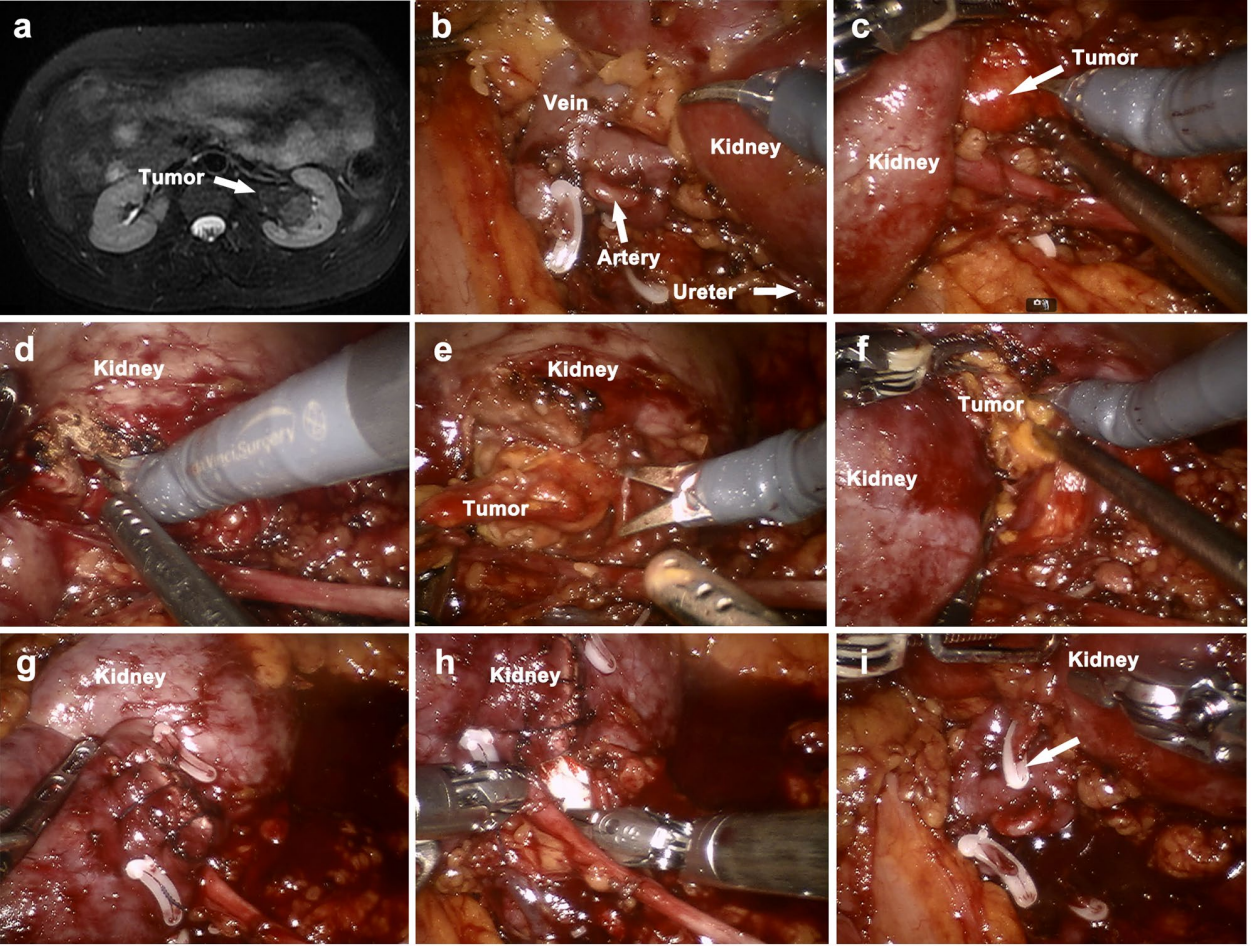
The MRI image of a typical RSAML and operation procedure (**a**) Preoperative MRI examination shows a typical RSAML of left kidney (**b**) Ureter and renal vessels are dissected separately. (**c**) RSAML is exposed (**d**) Renal labium is incised horizontally to get better tumor exposure (**e**) The outer part of RSAML is resected (**f**) The inner part of tumor is grasped, bluntly curetted and attracted (**g**) Renal labium is sutured with barbed thread (2 − 0) (**h**) The absorbable gelatin sponges are packed into renal sinus (**i**) Selective artery clipping is performed (white arrow)

From November 2018 to September 2022, 15 patients with RSAMLs who were treated with robotic NSS at the Department of Urology of Tongji hospital, were retrospectively included in the study. All the study patients’ RSAMLs were diagnosed with preoperative contrast-enhanced CT or MRI examination (Fig. 1a). RSAMLs were defined as those AMLs that arised from renal sinus. Inclusion criteria included large RSAMLs (> 8 cm), or RSAMLs with symptoms such as nausea, lumbar and abdominal pain, gross hematuria or hemorrhage. Patients with multiple lesions and tuberous sclerosis complex were excluded. Patients’ preoperative parameters, operative measures and postoperative outcomes were analyzed. Estimated glomerular filtration rate (eGFR) values were calculated using the Chronic Kidney Disease Epidemiology Collaboration (CKD-EPI) equation [14]. Perioperative complications were categorized according to the Clavien grading system [15]. Tumor complexity was evaluated using the RENAL nephrometry score [16].

### Surgical technique

All the operations were performed by one experienced surgeon (Dr. Guan Wei), using the da Vinci Si Surgical System. Robotic NSSs were performed via transperitoneal approach. Patients were positioned and trocars were placed as previously described [17].

The ureter and the renal vessels (renal vein and artery and their early branches) were individually dissected (Fig. 1b). The kidney was completely separated from perinephric fat. Dissection was performed along the edge of RSAML to expose the entire tumor (Fig. 1c). Then, renal ischemia was achieved by blocking the renal artery with a bulldog clamp, while the renal vein was left intact. A 1 cm incision was made horizontally in the renal labium to get better tumor exposure (Fig. 1d). The outer part of RSAML was resected along its base with curved scissors (Fig. 1e).

The inner part was grasped with ProGrasp forceps, and then bluntly curetted and attracted by a large aspirator until arrival at the pseudocapsule of AML and completely removal of RSAML surrounded by hilar vessels and collecting system (Fig. 1f). Pseudocapsule presented as reticular and slightly transparent capsule without obvious fat components. Small blood vessels supplying AML were bipolar electrocoagulated or clipped. A challenging dilemma is distinguishing AML from normal fat tissues surrounded by hilar vessels and collecting system. Usually, normal fat tissues were tightly attached to hilar vessels by connective tissues and could hardly be curetted and attracted. Fat tissues in AML had tougher texture, but were more fragile and easier to be attracted. Care should be taken to prevent damage to hilar vessels, collecting system and renal parenchyma when performing blunt curettage and aspiration.

Sutures of renal labium were performed with barbed thread (2 − 0) (Fig. 1g). The absorbable gelatin sponges were packed into renal sinus for compression hemostasis (Fig. 1h). Then the bulldog clamp was removed. In case of continued bleeding from sinus, selective artery clipping with hem-o-loc was performed (Fig. 1i). At last, specimen was taken out and one drainage tube was placed.

### Follow up

Patients came back to the hospital after one month to assess their postoperative recovery by examining the CT/ MRI and eGFR. In the follow-up, ultrasonography and eGFR were performed at postoperative 6 months, and then annually. CT was used when there was obvious shape change. Renal function preservation was defined as the ratio of eGFR at the latest follow-up to preoperative eGFR.

### Statistical analysis

Data were analyzed using SPSS 20.0 software. Mean ± standard deviation was used to present variables with normal distribution. Median [interquartile range] was used to present variables with non-normal distribution. The multiple comparison test was performed with one-way ANOVA.

## Results

Preoperative parameters, operative measures and postoperative outcomes of the study patients are summarized in Table 1. All the patients suffered lumbar and abdominal pain. Twelve (12/15, 80.00%) patients were women, and three (3/15, 20.00%) patients were men. Five (5/15, 33.33%) patients had history of abdominal surgery. Seven (7/15, 46.67%) patients underwent right-side robotic NSS, and eight (8/15, 53.33%) patients underwent left-side robotic NSS. The mean size of RSAMLs was 7.18 ± 2.51 cm. Among the study population, three patients underwent SAE at least 3 months prior to surgery, but showed no obvious reduction of tumor size. Two patients had ruptured AMLs 3 months prior to surgery: one patient received SAE and the other patient recovered with conservative treatment. The mean RENAL score was as high as 10 [10, 11].

Robotic NSS was successfully performed in all the patients, without any conversion to open surgery or nephrectomy. The mean operation time was 134.13 ± 40.56 min. The mean warm ischemia time was 25.73 ± 3.28 min. The median estimated blood loss was 100 [50, 270] ml. Of the 15 patients, one patient received intraoperative blood transfusion. The mean postoperative eGFR was 92.99 ± 20.14 ml/min/1.73m^2^. The mean drainage duration was 5.77 ± 1.98 days. The median postoperative hospital stay was 6.90 [5.80, 8.70] days. All the tumors were confirmed as AMLs pathologically. Two patients experienced postoperative urinary tract infection (Clavien-Dindo Grade II ), which were treated with antibiotic. No cases of urinary fistula, pneumothorax, wound infection or postoperative hemorrhage were detected.

Patients were followed-up for 25.53 ± 15.28 months. The latest follow-up eGFR recovered to 93.25 ± 17.30 ml/min/1.73m^2^, achieving 91.18% renal function preservation. There were no statistically difference among preoperative, postoperative and latest follow-up eGFR. During the follow-up period, no local recurrence occurred in all the patients.

**Table 1:** Preoperative parameters, operative measures and postoperative outcomes of the patients

Parameters		Value
Gender	Female, n(%)	12 (80.00%)
	Male, n(%)	3 (20.00%)
Age	Mean (years)	43.53 ± 12.48
BMI	(Kg/m^2^)	22.75 ± 2.81
Hypertension	n(%)	1 (6.67%)
History of abdominal surgery	n(%)	5 (33.33%)
History of prior SAE	n(%)	3 (20.00%)
Side of RSAML	Left, n(%)	8 (53.33%)
	Right, n(%)	7 (46.67%)
Size of RSAML	< 5 cm, n(%)	4 (26.67%)
	≥ 5 cm, n(%)	11 (73.33%)
	Mean (cm)	7.18 ± 2.51
History of rupture and bleeding	n (%)	2 (13.33%)
RENAL score		10 [10, 11]
ASA score		1.93 ± 0.26
Preoperative eGFR	(ml/min/1.73m^2^)	102.17 ± 15.67
Operation time	(min)	134.13 ± 40.56
Warm ischemia time	(min)	25.73 ± 3.28
Estimated blood loss	(ml)	100 [50, 270]
Transfusion	n (%)	1 (6.67%)
Postoperative eGFR	(ml/min/1.73m^2^)	92.99 ± 20.14
Drainage duration	(days)	5.77 ± 1.98
Postoperative hospital stay	(days)	6.90 [5.80, 8.70]
Complications	n (%)	2 (13.33%)
Follow-up time	(months)	25.53 ± 15.28
Latest follow-up eGFR	(ml/min/1.73m^2^)	93.25 ± 17.30
Recurrence	n (%)	0 (0%)

## Discussion

RSAML arises from renal sinus, accounting for quite a small part of renal AML. A growing tumor extends through renal hilum and outward into perinephric fat. RSAML is a typically complex renal tumor and often subjected to nephrectomy. With a high risk of long ischemia time, collecting system injury, and postoperative complications, NSS for centrally located renal tumors is technically demanding [18]. Even for experienced surgeons, it is quite a challenging work to perform NSS for these specially located tumors. Firstly, RSAML has a deep location, occupies renal sinus and compresses renal labium. There is little working space when handling the inner part. Secondly, renal vessels and collecting system are compressed by RSAML, resulting in abnormal hilar anatomy. Moreover, RSAML itself is enclosed by renal vessels and collecting system. Thirdly, due to RSAML’s fragility and thin tumor capsule, the tumor is easy to rupture and bleeding during the surgery procedure and leads to unclear operation field. In addition, spontaneous rupture results in giant retroperitoneal hematomas. Obvious tumor adhesion to adjacent organs is formed during the procedure of hematoma absorption, causing great difficulty in separating and exposing the tumor. In patients with highly complex renal tumors or AMLs, tumor complexity showed a good accuracy in predicting surgical outcomes and complications [19, 20]. Tumor size, the nearness to the urinary collecting system, and the involvement of renal sinus were the main predictors [19]. In our study, patients’ RENAL score was as high as 10, indicating that traditional partial nephrectomy may lead to poor surgical outcomes and high-grade complications. Therefore, a well-designed plan is critical in NSS for RSAML.

Here, we show modified robotic NSS as a safe, effective and feasible technique to treat RSAML. Our modified technique differs from traditional technique in two ways. Firstly, the inner part of tumor was grasped into pieces, to make curettage and aspiration more efficient and safer. Secondly, after removing the tumor, the parenchyma defect was not closed with sutures.

For AML, the goals of management are complete removal of the tumor, ameliorating symptoms, reducing risk of spontaneous rupture and renal function preservation as far as possible [21]. Robotic technology is being widely applied in renal surgery. Robotic surgery has the advantage of three-dimensional views, high quality images, flexible angle, and stable operation [22]. Compared with laparoscopic surgery, robotic partial nephrectomy may be a better option for treating large and complicated AML, as it can reduce warm ischemic time and preserve renal function more effectively [23]. Using minimally invasive technique, robotic NSS is performed successfully for malignant renal sinus tumors, but without more positive tumor margins or prolonged ischemia time [24]. Considering the complexity of RSAML, robotic NSS was planned. With clear view of operative field, renal vessels and tumor margins were easily determined. During dissection of the tumor, flexible angle and stable operation facilitated avoiding disruption of the tumor capsule, which could cause rupture and bleeding. During resection of the tumor, tumor among blood vessels and collecting system was accurately recognized and grasped into pieces.

The renal vessels and branches were dissected carefully. With sufficient exposure of them, the boundary between the tumor and the normal tissue was clearly identified, and the risk of injury and intraoperative blood loss were reduced and well-controlled. Injury to renal vessels leads to intraoperative and postoperative complications, such as bleeding and renal atrophy. Preoperative embolization could reduce blood loss and reduce tumor bulk during surgery [25, 26]. However, when confronted with AML arising from renal sinus, determining nutrient arteries of RSAML is difficult, as the direction of these vessels is parallel with that of renal hilar vessels. Therefore, we did not routinely perform a robotic NSS with preoperative embolization, but selectively clipped renal artery branch to reduce bleeding during the operation.

After blockage of renal artery and resection of the outer part of tumor, removal of the inner part in renal sinus was one the challenges of the operation. For skilled surgeons, a longitudinal incision is made along the Brodel line to remove malignant renal tumors. However, the total warm ischemia time is much longer [24]. AML is composed of varying proportions of fat tissues, smooth muscle, and blood vessels, which make it quite fragile. Previous studies indicate that laparoscopic curettage and aspiration could remove renal AML and avoid injury to the renal parenchyma and collecting system, especially for central renal AML [10, 27]. However, it was difficult to remove tumor among blood vessels and collecting system, in the condition of limited working space in renal sinus and abnormal hilar anatomy. Rough aspiration may cause mechanical injury. In our study, the inner part was bluntly grasped into pieces, to make curettage and aspiration more efficient and safer. These blunt procedures had little effect on renal artery and collecting system, as they had thick tube walls. However, venous wall was quite thin, and was prone to be injured. According to our experience, renal vein injury could be managed with sutures, to avoid excessive bleeding. In addition, this technique was an effective method of shortening the operation time, as it could fully expose small vessels at the base and stop bleeding points. A recent clinical study [28] reported that median warm ischemia time of robotic NSS in the treatment of central AML was only 21.50 min; this result is somewhat shorter than the warm ischemia time we reported, and we believe that this is mainly attributable to the much larger tumor size in our study(7.18 vs. 5.20 cm). Our single-center experience revealed the safety and effectiveness of the modified technique. We did not experience any conversion to open surgery or nephrectomy. One patient needed intraoperative blood transfusion. That surgery was performed at the beginning of our exploration of the modified robotic technique. No other intraoperative complications occurred.

Packing absorbable haemostat into the tumor bed and keep it tightly compressing parenchyma defect could achieve satisfied haemostasis [10]. In our study, several layers of absorbable gelatin sponges were applied and packed into the wound fossa, in order to reduce blood loss and drainage duration. The parenchyma defect was not closed with sutures, which may injure or obstruct renal vessels and collecting system. Compression and ischemic injuries to the renal parenchyma were avoided. The dosage of absorbable gelatin sponges depended on the size and depth of the wound fossa. Then total compression for a few minutes was carried out to ensure hemostasis. This procedure worked in case of small blood vessels injury or renal parenchymal hemorrhage. After the bulldog clamp was removed, renal hilum was inspected for hemostasis with low pneumoperitoneum pressure. Continued bleeding from sinus may be attributed to injury of tumor’s nutrient artery. Based on bleeding location, suspicious renal artery branch was clamped experimentally. When it did work, selective artery clipping was performed. The intraoperative blood loss was comparable to previous studies of surgery strategies for AML [6, 13] and no postoperative hemorrhage was detected, demonstrating that strategy for hemostasis was effective.

There are also some limitations in our study, including lack of control group, its retrospectively nature, small sample size and limited follow-up period. All the patients are treated in a single center, and a further prospective multi-center study is needed.

## Conclusions

Robotic NSS for RSAML is a complicated procedure that demands technical expertise and a well-designed strategy is critical in the operation. Treating RSAML with modified robotic NSS is safe, effective and feasible.

## Data Availability

The data that support the findings of this study are available from the corresponding author, Fan Li and Wei Guan, upon reasonable request.

## Abbreviations

RSAML: Renal sinus angiomyolipoma
NSS: Nephron-sparing surgery
eGFR: Estimated glomerular filtration rate
AML: Angiomyolipoma
SAE: Selective arterial embolization
